# Respiratory viral pathogens among Singapore military servicemen 2009 – 2012: epidemiology and clinical characteristics

**DOI:** 10.1186/1471-2334-14-204

**Published:** 2014-04-15

**Authors:** Xin Quan Tan, Xiahong Zhao, Vernon J Lee, Jin Phang Loh, Boon Huan Tan, Wee Hong Victor Koh, Sock Hoon Ng, Mark I-Cheng Chen, Alex Richard Cook

**Affiliations:** 1 Biodefence Centre, Ministry of Defence, Singapore, Singapore; 2 Preventive Medicine Residency Programme, National University Health System, Singapore, Singapore; 3 Saw Swee Hock School of Public Health, National University of Singapore, Singapore, Singapore; 4 Defence Medical and Environmental Research Institute, Singapore, Singapore; 5 Department of Clinical Epidemiology, Tan Tock Seng Hospital, Singapore, Singapore; 6 Yale-NUS College, National University of Singapore, Singapore, Singapore; 7 Program in Health Services and Systems Research, Duke-NUS Graduate Medical School, Singapore, Singapore; 8 Department of Statistics and Applied Probability, National University of Singapore, Singapore, Singapore

**Keywords:** Influenza like-illness-ILI, Surveillance, Epidemiology, Influenza, Respiratory infections, Respiratory viruses

## Abstract

**Background:**

Few studies have comprehensively described tropical respiratory disease surveillance in military populations. There is also a lack of studies comparing clinical characteristics of the non-influenza pathogens with influenza and amongst themselves.

**Methods:**

From May 2009 through October 2012, 7733 consenting cases of febrile respiratory illness (FRI) (temperature [greater than or equal to]37.5degreesC with cough or sorethroat) and controls in the Singapore military had clinical data and nasal washes collected prospectively. Nasal washes underwent multiplex PCR, and the analysis was limited to viral mono-infections.

**Results:**

49% of cases tested positive for at least one virus, of whom 10% had multiple infections. 53% of the FRI cases fulfilled the definition of influenza-like illness (ILI), of whom 52% were positive for at least one virus. The most frequent etiologies for mono-infections among FRI cases were Influenza A(H1N1)pdm09 (13%), Influenza B (13%) and coxsackevirus (9%). The sensitivity, specificity, positive predictive value and negative predictive value of ILI for influenza among FRI cases were 72%, 48%, 40% and 69% respectively. On logistic regression, there were marked differences in the prevalence of different symptoms and signs between viruses with fever more prevalent amongst influenza and adenovirus infections than other viruses.

**Conclusion:**

There are multiple viral etiologies for FRI and ILI with differing clinical symptoms in the Singapore military. Influenza and coxsackevirus were the most common etiology for FRI, while influenza and adenoviruses displayed the most febrile symptoms. Further studies should explore these differences and possible interventions.

## Background

Influenza-like illness (ILI) is often used for influenza surveillance [1], as influenza is a disease of global interest with 5% of adults developing symptomatic disease annually and with case fatalities of 3.5% in susceptible populations [2]. While influenza surveillance remains a priority, ILI can also be caused by a wide range of viral pathogens that present with a spectrum of respiratory symptoms [3-6]. In the tropics, viral respiratory pathogens have been reported to exhibit different seasonality and transmission characteristics compared to temperate climates [2,7-9]. This necessitates a better understanding of their epidemiology to assess the utility and importance of surveillance in these settings. The year-round circulation of respiratory viruses in the tropics may also predispose patients to co-infection with multiple pathogens, with implications for severity of disease [10,11] and secondary bacteria infection [12,13].

While there have been studies comparing differences in clinical presentation between influenza and non-influenza cases [14], few describe the epidemiology and differences in clinical presentation among various non-influenza respiratory viruses. As influenza viruses have accounted for only between 10.1% to 53.0% of all ILI cases [15-17], it is important to understand the contribution of other respiratory pathogens to overall morbidity and to determine their epidemiological distribution and clinical presentation.

To address these issues, this study explores data obtained from a respiratory disease sentinel surveillance system in the Singapore military to examine the etiologic viral agents of respiratory illnesses in a tropical environment, to determine the viruses that circulate post-influenza vaccination, and to compare the differences in clinical presentation.

## Methods

### Study site and population

Singapore is a city-state in tropical South-East Asia with a population of 5.3 million people (mid-year 2012). The Singapore military is based on national service in which all male Citizens and liable Permanent Residents serve for two years after high school. Servicemen typically live in barracks-style accommodation on weekdays and return home on weekends.

The Singapore military started a sentinel respiratory disease surveillance program in 5 major camps (including a recruit training camp) on 11 May 2009, tracking febrile respiratory illness (FRI) cases (temperature ≥37.5°C with cough or sore throat). The definition of FRI contrasts with influenza-like illness (ILI, defined as fever ≥38.0°C with cough or sore throat) to broaden the capture of other febrile cases that also result in absenteeism while limiting cases to those with fever as an indicator of severity. This allows for detection of a larger number of respiratory pathogens.

Patients who visited the primary healthcare clinics in the camps between 11 May 2009 and 31 October 2012 during regular consultation hours who met the FRI criteria were recruited. Healthcare workers obtained written informed consent, administered a questionnaire, obtained clinical specimens and performed a clinical examination on partcipants. Repeat consultations were excluded if the healthcare worker determined that the patient had not recovered from the first illness episode. We also obtained samples from controls (those without respiratory symptoms or acute infections), who were recruited across the year at between 5 to 10 persons per week. Informed consent, the baseline questionnaire, and clinical specimens were obtained.

From December 2009, all recruits were administered with the Influenza A(H1N1)pdm09 (FLU-A(H1N1)pdm09) vaccine. The trivalent seasonal influenza vaccination was first introduced to recruits in December 2010, followed henceforth by all other personnel in November 2011.

### Laboratory methods

Nasal washes from each side of the nose were taken from consenting participants by trained medical staff, placed in viral transport media and refrigerated. The samples were transported to the laboratory on ice for etiological testing within 24 hours.

Laboratory analysis was performed in an ISO15189-accreditated laboratory for molecular diagnostics which regularly takes part in external proficiency programs such as QCMD EQA programs. Detailed laboratory methods were previously described [14]. We used the multiplex PCR strategy based on the Resplex assays described below, and performed additional singleplex PCR assays to determine the influenza subtype. Total nucleic acids were extracted from each specimen using the DNA minikit (Qiagen, Inc, Valencia, CA, USA) according to manufacturer’s instructions. A total of 20 μl of extract were tested with Resplex I and II (version 2.0, Qiagen, Inc., Valencia, CA, USA) [18] for respiratory micro-organisms on the LiquiChip 200 Workstation, according to manufacturer’s instructions. The Resplex I and II (version 2.0) assays are multiplex PCR assays coupled with bead array detection technology and can simultaneously detect and subtype 18 different pathogens including influenza A (FLU-A) and influenza B (FLU-B). Specimens that were Resplex II positive for FLU-A were further subtyped with real-time PCR for H1 or H3 (Singapore Ministry of Health), or for FLU-A(H1N1)pdm09. Briefly, 5 μl of total genetic extracts were tested using an in-house developed assay based on the one-step SuperscriptIII/Platinum Taq kit (Invitrogen, Carlsbad, CA, USA) following manufacturer’s instructions on the LightCycler machine from Roche or the Applied Biosystems real-time PCR machine (7500).

### Statistical methods

The analysis was limited to viral mono-infections amongst cases to discern clinical presentations and symptom complexes associated with each pathogen. We excluded viruses with fewer than 20 cases (0.6% of the total), as the number was too small to have a reasonable sample size – these were Coronavirus HKU1 (CoV-HKU1), Parainfluenza 1 (hPIV-1), hPIV-2, hPIV-4, Influenza A(H1N1) (the prepandemic strain), respiratory syncytial virus A (RSV A), RSV B, CoV and Bocavirus (BV). This left 14 viruses for the subsequent analyses.

The main aim was to compare the differences in clinical expressions, including individual symptoms (or signs), pairs of symptoms, and overall symptom load between patients with different viral infections. We counted the clinical symptoms/signs and calculated the corresponding empirical proportions with 95% confidence intervals (CIs) to evaluate the overall symptom load. Logistic regression analysis was used to investigate the differences in symptom expressions for each pair. Differences were identified at a significance level of 0.05. To assess the presence of paired symptoms/signs for all viruses, we conducted binomial tests to compare the joint proportions of symptom pairs occurring together to the expected proportions assuming independence of symptoms. The ratio of the observed proportion of symptom pairs relative to the product of the marginal proportion of each symptom is defined as the excess probability ratio which measures effect size. Multivariate logistic regression analysis was performed to compare the risk of having an individual symptom/sign among viral mono-infections by assigning a categorical variable for all viruses as the primary predictor. Potential confounding was addressed by adjusting the model for age, smoking status, asthma and heart disease. Non-significant variables were dropped at a significance level of 0.05 to obtain the final model. Statistical analyses were performed using the R Statistical Software (version 3.0.0) [19].

Ethics approval was given by the Singapore military’s Joint Medical Committee for Research, and the National University of Singapore’s ethics review committee.

## Results

The basic demographic data are described in Table 1. Participants were mostly young male adults, with other characteristics largely similar. However, there were significantly less recruits amongst controls than amongst other groups.

**Table 1:** Demographics of all participants, cases, controls and mono-infection cases

**Characteristics**	**All (Count, %)**	**Cases (Count, %)**	**Controls (Count, %)**	**Mono-infection (Count, %)**	**p-value†**
**Age (Mean, SD)**	20.8 (3.1)	20.7 (3.1)	21.1 (2.9)	20.7 (3.2)	0.781
**Male**	9055 (99.8)	7713 (99.7)	1342 (99.9)	3422 (99.8)	0.902
**Current Smoker**	2475 (27.3)	2090 (27.0)	385 (28.6)	887(25.9)	0.220
**Recruit**	3870 (42.6)	3627 (46.9)	243 (18.1)	1575 (45.9)	<0.001
**Asthma**	1833 (20.2)	1597 (20.7)	236 (17.6)	721 (21.0)	0.051
**Heart Disease**	102 (1.1)	83 (1.1)	19 (1.4)	37 (1.1)	0.748
**Total**	9077 (100.0)	7733 (100.0)	1344 (100.0)	3430 (100.0)	-

† By ANOVA test, comparing mean age, and by Pearson's chi-square test, comparing proportions across all categories.

**Figure 1:**
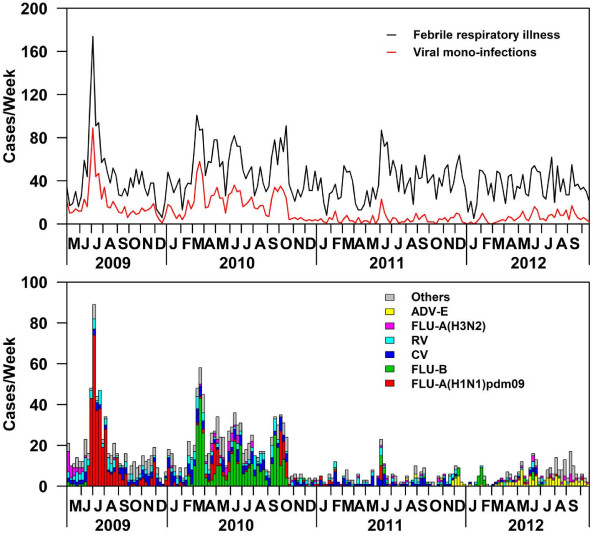
Distribution of weekly cases of febrile respiratory illness (FRI), viral mono-infections during study period*. *The top panel presents the weekly FRI cases together with viral mono-infection cases. The second panel is a frequency chart presenting the weekly viral mono-infection cases. The dominating virus was placed at the bottom of each bar. Viruses are shaded in different colors – Adenovirus E (ADV-E); Influenza A(H3N2) (FLU-A(H3N2)); Rhinovirus (RV); Coxsackie/Echovirus (CV); Influenza B (FLU-B) and influenza A(H1N1)pdm09 (FLU-A(H1N1)pdm09). Influenza A(H1N1) (FLU-A(H1N1) and Influenza (unknown type) (FLU-A(unknown)), Adenovirus B (ADV-B) and ADV(untyped), Enterovirus (EV), human metapneumovirus (hMPV), Parainfluenza 1 (hPIV-1), hPIV-2, hPIV-3 and hPIV-4,Coronavirus OC43 (CoV-OC43), CoV-NL63, CoV-229E, CoV-HKU1 and CoV(untyped), respiratory syncytial virus A (RSV-A) and RSV-B and Bocavirus (BV) are pooled as others in the bottom panel.

The temporal distribution of cases is described in Figure 1. No obvious overall seasonal pattern can be observed. The peak in June and July 2009 corresponds to the FLU-A(H1N1)pdm09 pandemic [16]. As this peak tailed off, we observed an increase in FLU-B cases (starting Feb–Mar 2010). Subsequently, as the FLU-B cases fell, Adenovirus E (ADV-E) cases started to increase. Coxsackie/echovirus (CV) and rhinovirus (RV) infections were consistently present in the earlier periods but appeared to tail off by 2012, corresponding to the rise in FRI cases due to other viruses.

The etiologies of selected infections are illustrated in Table 2. At least one virus was detected in 3794 of the 7733 FRI cases (49.1%). In 376 (9.0%) of the 3794 cases, more than one virus was detected and these were excluded. 4120 (53.3%) of the FRI cases fulfilled the definition of ILI; 2146 (52.1%) of these ILI cases were positive for at least 1 virus. Of the 3430 FRI mono-infection cases, 2128 (62.0%) were viral. 1259 (59.2%) of these 2128 cases met the definition of ILI.

**Table 2:** Etiology of by cases, controls and mono-infections

	**FRI Cases (n = 7733)**		**Controls (n = 1344)**	**Mono-infection (n = 3430†)**
**Virus**	**No. of positives (%) (n = 3794)**	**No. of ILIs positives (%) (n = 2146)**	**No. of positives (%) (n = 115)**	**No. of positives (%) (n = 2128)**
**Coxsackie/Echovirus**	708 (9.2)	318 (4.1)	37 (2.8)	324 (9.4)
**Influenza B**	604 (7.8)	449 (5.8)	7 (0.5)	441 (12.9)
**Rhinovirus**	574 (7.4)	220 (2.8)	24 (1.8)	257 (7.5)
**Influenza A(H1N1)pdm09**	568 (7.3)	393 (5.1)	3 (0.2)	459 (13.4)
**Adenovirus E**	516 (6.7)	317 (4.1)	9 (0.7)	100 (2.9)
**Coronavirus OC43**	235 (3.0)	103 (1.3)	10 (0.7)	83 (2.4)
**Human Metapneumovirus**	142 (1.8)	82 (1.1)	0 (0.0)	60 (1.7)
**Influenza A(H3N2)**	137 (1.8)	96 (1.2)	4 (0.3)	107 (3.1)
**Parainfluenza 3**	122 (1.6)	58 (0.8)	1 (0.1)	48 (1.4)
**Adenovirus B**	116 (1.5)	85 (1.1)	4 (0.3)	29 (0.8)
**Enterovirus**	109 (1.4)	42 (0.5)	4 (0.3)	35 (1.0)
**Coronavirus NL63**	62 (0.8)	31 (0.4)	0 (0.0)	34 (1.0)
**Coronavirus 229E**	61 (0.8)	26 (0.3)	6 (0.4)	30 (0.9)
**Influenza A (Unknown type)**	60 (0.8)	34 (0.4)	8 (0.6)	41 (1.2)

The following pathogens recorded less than 20 mono-infection cases each and were not included in the table: Coronavirus HKU1, Parainfluenza 1, 2 and 4, Influenza A/H1, Respiratory Syncytial Virus A and B, Bocavirus.

† Of the 3430 mono-infection cases, there are 1904 ILI cases.

We examined the proportion of ILI cases among those with viral mono-infections (Table 3). Influenza viruses accounted for only 40% of ILI. Among FRI cases, more than 60% of patients with influenza and adenovirus infections presented with ILI. However, several other viral infections led to high rates of ILI, including CV, Human Metapneumovirus (hMPV) and CoVs. The sensivity, specifity, positive predictive value (PPV) and negative predictive value (NPV) of ILI for influenza was 72.2%, 48.1%, 40.1% and 69.3% respectively.

**Table 3:** Summary of virus mono-infections having influenza-like illness (ILI), 11 May 2009 to 31 Oct 2012

**Virus**	**Probability of virusgiven ILI (%) (95% CI)**	**Probability of having ILIgiven infection, by virus (%) (95% CI)**
Influenza A(H1N1)pdm09	16.7 (15.1 to 18.5)	69.3 (64.8 to 73.5)
Influenza A(H1N1)	0.4 (0.2 to 0.8)	80.0 (44.4 to 97.5)
Influenza A(H3N2)	4.2 (3.4 to 5.2)	74.8 (65.5 to 82.7)
Influenza A(Unknown type)	1.4 (0.9 to 2.0)	63.4 (46.9 to 77.9)
Influenza B	17.4 (15.8 to 19.2)	75.3 (71.0 to 79.2)
Coxsackie/Echovirus	7.9 (6.7 to 9.2)	46.6 (41.0 to 52.2)
Enterovirus	0.5 (0.2 to 0.9)	25.7 (12.5 to 43.3)
Rhinovirus	5.1 (4.2 to 6.2)	37.7 (31.8 to 44.0)
Adenovirus B	1.4 (0.9 to 2.0)	89.7 (72.7 to 97.8)
Adenovirus E	3.4 (2.6 to 4.3)	65.0 (54.8 to 74.3)
Adenovirus	0.1 (0.0 to 0.3)	50.0 (1.3 to 98.7)
hMPV	1.9 (1.3 to 2.6)	60.0 (46.5 to 72.4)
Coronavirus 229E	0.6 (0.3 to 1.1)	40.0 (22.7 to 59.4)
Coronavirus HKU1	0.4 (0.2 to 0.8)	41.2 (18.4 to 67.1)
Coronavirus NL63	0.8 (0.5 to 1.4)	47.1 (29.8 to 64.9)
Coronavirus OC43	1.7 (1.2 to 2.4)	38.6 (28.1 to 49.9)
Coronavirus	0.0 (0.0 to 0.2)	0.0 (0.0 to 97.5)
Others†	2.3 (1.6 to 3.0)	43.9 (33.9 to 54.3)

Non-viral aetiologies accounted for 33.9% of the ILIs.

† RSV A, RSV B, Parainfluenza 1, Parainfluenza 2, Parainfluenza 3, Parainfluenza 4, and Bocavirus are pooled as others.

On univariate comparison of clinical symptoms and signs, Adenovirus B (ADV-B), as well as Influenza A(H3N2) (FLU-A(H3N2)), FLU-A(H1N1)pdm09 and FLU-B tended to cause fever more frequently (0.31 (95% CI: 0.14,0.48), 0.30 (0.21,0.40), 0.23 (0.17,0.28), 0.28 (0.22,0.33)). ADV-E was inclined to cause less respiratory symptoms (cough with phlegm, dry cough, running nose)(-0.08 (-0.17, 0.03), -0.26 (-0.37, -0.16), -0.18 (-0.28, -0.07)).

**Figure 2:**
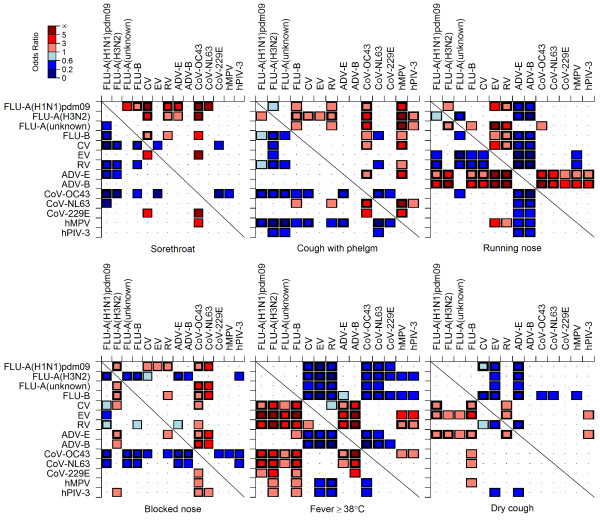
**Multivariate analysis comparing viruses among clinical features adjusted for age, smoking status.** Age, smoking status and a categorical predictor for viruses were included in the analysis before subsequently removing non-significant variables. Columns represent the categorical predictor for viruses, and each row corresponds to a virus that was chosen as the reference group. The viruses included from the top row to the bottom row are Influenza A(H1N1)pdm09 (FLU-A(H1N1)pdm09), Influenza A(H3N2) (FLU-A(H3N2)), Influenza A(unknown type) (FLU-A(unknown), Influenza B (FLU-B), Coxsackie virus (CV), Enterovirus (EV), Adenovirus E (ADV-E), ADV-B, Coronavirus OC43 (CoV-OC43), CoV-NL63, CoV-229E, Human Metapneumovirus (hMPV) and Parainfluenza 3 (hPIV-3). Color cells represent variables that are significant at the 5% level, and the thickness of the cell wall represents the p-value (thin means 0.01 < p < 0.05; medium, 0.001 < p < 0.01; and thick, p < 0.001). The odds ratios are encoded by colors where a red cell indicates an odds ratio > 1; and blue otherwise. For example, for a sore throat, FLU-A(unknown), FLU-B, CV, RV, ADV-E, CoV-OC43 and CoV-NL63 have more of the sore throat than IFLU-A(H1N1)pdm09 indicated by the red cells in the row for FLU-A(H1N1)pdm09 and corresponding columns.

From the multivariate analysis (Figure 2), compared to most other viruses, A FLU-A(H1N1)pdm09 and FLU-A(H3N2) less commonly resulted in sorethroat. Running nose was more common in enterovirus (EV) and RV cases and less common in ADV. FLU-B was more likely than a majority of the other viruses to cause dry cough while EV and ADV-E were less likely to cause dry cough. CoV-OC43 and hMPV were more likely to cause cough with phlegm than the most other viruses. Influenza viruses and adenoviruses were more likely to cause fever ≥38.0°C.

**Figure 3:**
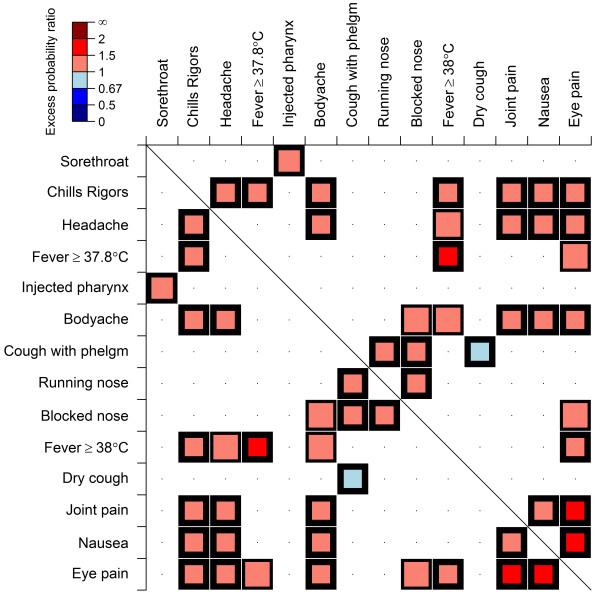
**Correlation of Symptoms and Signs Across all Viruses.** Clinical signs or symptoms are listed by average frequency from the most to the least. Binomial test is used to assess the discrepancy between the observed proportion of symptom pairs and the expected proportion of symptom pairs which is the product of the two marginal distributions by assuming symptoms develop independently. Color cells represent differences that are significant at the 5% level, and the thickness of the cell wall represents the p-value (thin means 0.01 < p < 0.05; medium, 0.001 < p < 0.01; and thick, p < 0.001). The excess probability encoded by colors measures the effect size. If the observed proportion is lower than the expected proportion, the cell will be shaded by blue color, and red color otherwise.

In Figure 3, we explored the associations (and dissociations) between different clinical symptoms and signs across all viruses. Some are expected, such as association of fever ≥37.8°C and fever ≥38.0°C and dissociation of dry cough and cough with phlegm. Fever ≥38.0°C was also associated with systematic complaints, such as chills, bodyache, headache and eye pain. Sorethroat was associated with an injected pharynx.

## Discussion

Our study shows the different viral etiologies of ILI and compares the clinical characteristics of different viral etiologies in a tropical setting. This data series only spanned three years, and initial observations showed no clear seasonal variation compared to temperate regions, similar to previous reports of overall tropical respiratory disease patterns [7,9,20]. The initial peak corresponded to the FLU-A(H1N1)pdm09 pandemic [21], with the subsequent lower incidence in the recruit population likely due to vaccination with the pandemic vaccine a year before annual seasonal vaccination was started across all personnel [22].

The number of FRI cases remained fairly consistent throughout the study period (except the pandemic). However, prevalence of pathogens varied throughout, with some negative correlation observed between the viruses – e.g. a drop in FLU-A followed by a rise in FLU-B activity, and a drop in FLU-B cases followed by a rise in adenovirus activity. Correlation of viral activity have previously been reported – Wang et al [23] reported negative association between RV and ADV rates, while Bellei et al [15] and Razanajatovo et al [24] described concomitant rise of influenza and RV, and influenza and ADV activity respectively. In addition, Kasper et al reported that ILI rates remained constant despite varying prevalences of influenza [25]. This supports our findings that multiple agents are capable of causing ILI, and a decrease in the prevalence of one virus was replaced by an increase in prevalence of another. Further studies across a longer time period are necessary, especially for vaccine effectiveness evaluation.

49.1% of FRI and 52.1% of ILI cases were positive for a virus, similar to the 44.5% and 61.8% reported by studies targeting similar panel of organisms [3,6,15,17,26,27]. The remaining FRI cases may be due to non-viral agents, agents beyond the ability of the test, non-infectious causes, and possible sampling errors. Viruses most commonly detected in ILI cases were FLU-A, FLU-B, and CV in that order. Influenza was also the top etiologic agent for ILI in some studies [6,15,17,24,28] although other pathogens have been identified to be most prevalent in different settings, such as RSV and hMPV in France [29], influenza and RSV in the USA [30], influenza and RV in Central America [16] and China [6] and in Italy, influenza and ADV [31]. The range of pathogens indicates a need to perform local continual surveillance since prevailing pathogens differ across different populations, geographic regions and climates. The high incidence of CV warrants further study – hand, foot and mouth disease is endemic to Singapore and CV is frequently identified in pediatric samples [32], and it is possible that CV circulates at high levels in adults also.

Previous studies have identified a co-infection rate of 11.0 to 47.0% [6,11,24,33,34] for viruses, higher than the 9.9% found in this study despite some studies using a less extensive diagnostic panel in a similar age group. Possible reasons include our highly influenza-vaccinated population or a warmer climate with higher relative humidity resulting in lower virus circulation [35] and a study population that did not include children (studies reported higher co-infection rates amongst pediatric patients [17,24]).

In both univariate and multivariate analysis, ADV and influenza viruses were more likely to cause fever (≥38.0°C). This finding has been demonstrated in other studies [14,15,36-39]. Fever also tends to be associated with other systemic complaints such as eye pain, bodyache and headache; this may be due to cytokine mediated systematic inflammatory response [40] and could indicate more severe disease. Bellei et al’s [15] study in Brazil also found that EV, RV and CoV were least likely to cause fever, and that RV and EV cases were the most likely to present with rhinorrhea. Cough with sputum in FLU-A(H3N2) and FLU-A were less prevalent than in other viruses, in contrast to reports in other settings [14,37-39]. We found that FLU-B cases were more likely to report dry cough, similar to other studies [14,37-39]. The heterogeneity of results across different studies highlight the difficulty of using clinical symptoms in determining the etiology of ILI. We also detected a small proportion of asymptomatic individuals who tested positive for the various viruses (Table 2). These could represent carriage without infection or subclinical/asymptomatic infections during periods of virus circulation.

Although ILI is widely used to identify influenza, the traditional definition would have picked up only 69% of influenza infections (except untyped FLU-A) that were identified as FRI. ADV infections also frequently fulfilled the ILI definition (89.7% of ADV-B and 65.0% of ADV-E), as did substantial fractions of CV, hMPV, and CoV. Only 40.1% of viral mono-infections that met the ILI definition were due to influenza. We found a fairly high sensitivity (72.2%) of ILI for influenza, but a low specificity (48.1%) in keeping with sensitivities of 55.4–86.8% and specifities of 39.3–67% reported elsewhere [25,39,41,37]. PPV of ILI was low (40.1%) compared to other studies (77.6–79.0%) likely because these studies were conducted during influenza seasons. This is supported by the higher NPV (69.3%) compared to other studies (48.9–55.0%) [25,39,41,37]. Two tropical studies [14,25] also report low PPV of 21.0–37% and high NPV of 81–90.0%.

ADV are as likely as influenza viruses to present with fever and tend to be captured by the ILI definition. However, they are more likely to present with cough and sorethroat than influenza. It may be possible to differentiate ADV and influenza infection based on rhinorrhea and other symptoms. This could be useful in surveillance and clinical management, especially when deliberating whether to start antivirals. Early etiologic diagnosis of influenza has been shown to be cost effective [42] with reduced antibiotic use and may reduce complications with early antivirals. It may be possible to combine a clinical diagnostic model with rapid testing to achieve these goals.

### Limitations

The analysis was limited to viral mono-infections and future studies should explore co-infections and bacterial infections. This study involved predominantly young adult males, and results may not be generalizable to the overall population, necessitating further studies among various age groups and gender. There were also less recruits amongst controls than amongst other groups, and this would be an important consideration when comparing the two groups in the future. Finally, the actual clinical impact of differentiating between various viral etiological agents may be limited, and we could not determine the relative severity of symptoms other than fever.

## Conclusion

Our study highlights the varied etiology for FRI and ILI in the tropical setting – influenza and ADV and CV were all common. Influenza and ADVs tend to present with higher fever, and vaccination should be considered. The utility of ILI for tropical surveillance of influenza needs to be reviewed given the low PPV and high NPV compared to temperate regions. The surveillance system has enabled the Singapore military to understand the etiologic agents affecting servicemen, hence implementing and evaluating controls measures such as vaccination.

## Competing interests

VJL had previously received unrelated research grants from GSK.

## Authors’ contributions

XQT wrote the manuscript. XZ analysed the data and assisted with the writing of the manuscript. ARC and MIC revised the manuscript and assisted with data analysis. VL conceptualized the study and revised the manuscript. BHT, JPL, WHVK, SHN were involved in the laboratory testing of the specimens. All authors read and approved the final manuscript.

## Pre-publication history

The pre-publication history for this paper can be accessed here:

http://www.biomedcentral.com/1471-2334/14/204/prepub
