# Erratum To: Respiratory viral pathogens among Singapore military servicemen 2009 – 2012: epidemiology and clinical characteristics

**DOI:** 10.1186/1471-2334-14-326

**Published:** 2014-07-24

**Authors:** Xin Quan Tan, Xiahong Zhao, Vernon J Lee, Jin Phang Loh, Boon Huan Tan, Wee Hong Victor Koh, Sock Hoon Ng, Mark I-Cheng Chen, Alex Richard Cook

**Affiliations:** Biodefence Centre, Ministry of Defence, Singapore, Singapore; Preventive Medicine Residency Programme, National University Health System, Singapore, Singapore; Saw Swee Hock School of Public Health, National University of Singapore, Singapore, Singapore; Defence Medical and Environmental Research Institute, Singapore, Singapore; Department of Clinical Epidemiology, Tan Tock Seng Hospital, Singapore, Singapore; Yale-NUS College, National University of Singapore, Singapore, Singapore; Program in Health Services and Systems Research, Duke-NUS Graduate Medical School, Singapore, Singapore; Department of Statistics and Applied Probability, National University of Singapore, Singapore, Singapore

## Text

A minor error was found in Table 1 that described and compared the demographics amongst all participants, cases, controls and mono-infections after publishing this work [1]. For recruits, the number and the corresponding proportion in each group was under-reported. This was caused by discrepancies in the database in how recruits were coded that were not noticed until now. We have recoded recruits in the database and have re-analysed data. The differences in proportions of recruits amongst all groups remained significant with the revised data (Table 1). These errors only affect this table. All the other results and conclusions remain.

**Table 1:** **Demographics of all participants, cases, controls and mono-infection cases**

Characteristics	All (Count,%)	Cases (Count,%)	Controls (Count,%)	Mono-infection (Count,%)	p-value†
**Age (Mean, SD)**	20.8 (3.1)	20.7 (3.1)	21.1 (2.9)	20.7 (3.2)	0.781
**Male**	9055 (99.8)	7713 (99.7)	1342 (99.9)	3422 (99.8)	0.902
**Current Smoker**	2475 (27.3)	2090 (27.0)	385 (28.6)	887(25.9)	0.220
**Recruit**	6048 (66.6)	5568 (72.0)	480 (35.7)	2495 (72.7)	<0.001
**Asthma**	1833 (20.2)	1597 (20.7)	236 (17.6)	721 (21.0)	0.051
**Heart Disease**	102 (1.1)	83 (1.1)	19 (1.4)	37 (1.1)	0.748
**Total**	9077 (100.0)	7733 (100.0)	1344 (100.0)	3430 (100.0)	-

† By ANOVA test, comparing mean age, and by Pearson’s chi-square test, comparing proportions across all categories.

We apologise for this oversight, and the inconvenience that we might have brought.
